# Economic burden of short-acting beta-2 agonist (SABA) overuse among asthma patients in Türkiye: a cost analysis with respect to the updated GINA treatment recommendations

**DOI:** 10.1186/s12890-024-03327-9

**Published:** 2024-10-21

**Authors:** Arzu Yorgancıoğlu, Kurtuluş Aksu, Ceyhun Cura, Yiğit Yaman, Melda Dinç, Simten Malhan, the SABINA Türkiye Study Group*, Deniz Kızılırmak, Nejat Altıntaş, İsmet Bulut, Tülin Çağatay, Bilun Gemicioğlu, Özgür İnce, Kıvılcım Oğuzülgen, Dilşad Mungan, Füsun Kalpaklıoğlu, Ayşe Baççıoğlu, Funda Aksu, Murat Altuntaş, Ferda Öner Erkekol, Gül Karakaya, Ali Fuat Kalyoncu, Ebru Damadoğlu, İsmail Hanta, Ersoy Altunok, Adviye Özer, Sibel Atış Naycı, Demet Polat Yuluğ, Gazi Gülbaş, Mecit Süerdem, Burcu Yormaz, Emel Ceylan, Duygu Erge, Aykut Çilli, Berat Celil Doğan, Fuat Erel, Can Sevinç, Ceyda Anar, Dane Ediger, Gülseren Pekbak, Müge Erbay

**Affiliations:** 1 Department of Pulmonology, Faculty of Medicine, Manisa Celal Bayar University, Manisa, Türkiye; 2 Division of Immunology and Allergy, Department of Chest Disease, University of Health Sciences, Ankara Atatürk Sanatoryum Training and Research Hospital, Ankara, Türkiye; 3 AstraZeneca, İzmir, Türkiye; 4 AstraZeneca, İstanbul, Türkiye; 5 Department of Healthcare Management, Faculty of Health Sciences, Başkent University, Ankara, Türkiye

**Keywords:** Adult asthma, Asthma severity, GINA updates, SABA overuse, Cost analysis, Cost burden, Direct costs, Türkiye

## Abstract

**Background:**

This cost of illness study aimed to determine economic burden of short-acting β2-agonist (SABA) overuse in Türkiye from payer perspective with respect to the updated GINA 2022 treatment recommendations.

**Methods:**

A total of 3,034,879 asthma patients comprised the study population, via estimations extrapolated from the Türkiye arm of the global SABINA III study. The economic burden (costs related to the drug use and severe exacerbations) was compared in subgroups of overall (≥ 0 canisters/year) vs. GINA-recommended (0–2 canisters/year, hypothetical population) SABA use and in subgroups of appropriate use (0–2 canisters/year, real population) vs. overuse (≥ 3 canisters/year) of SABA with extrapolation of SABINA Türkiye data to the Türkiye asthma population.

**Results:**

Recommended SABA use was predicted to prevent 127,505 of 157,512 severe exacerbations per year in mild asthma patients and 2,668,916 of 3,262,800 severe exacerbations per year in moderate-severe asthma patients. Annual cost burden of not applying recommended SABA use (overall [≥ 0 canisters/year] vs. GINA-recommended [0–2 canisters/year] SABA use) in mild asthma and moderate-severe asthma patients was calculated to be €20.43 million and €427.65 million in terms of severe exacerbations, and to be €829,352 and €7.20 million in terms of drug costs, respectively. The total annual economic burden arising from not applying recommended SABA use was estimated to be €456.11 million. Appropriate use (0–2 canisters/year) vs. overuse (≥ 3 canisters/year) of SABA was associated with decreased frequency of severe exacerbations per year in mild asthma (from 129,878 to 27,634) and moderate-severe asthma (from 2,834,611 to 428,189) patients. SABA overuse in mild and moderate-severe asthma patients was estimated to yield an additional annual cost of €16.38 million and €385.59 million, respectively in terms of severe exacerbations, and a total €11.30 million additional drug cost. The overall annual economic burden arising from SABA overuse was estimated to be €413.27 million.

**Conclusions:**

The estimated annual total economic burden arising from not applying recommended SABA use (€456.11 million) and SABA overuse (€413.27 million) with respect to the updated GINA 2022 treatment recommendations indicates the substantial cost burden of SABA overuse to the Turkish National Health System, corresponding up to 26% of the total direct cost of asthma reported in our country.

## Introduction

Asthma is a chronic inflammatory respiratory disease with a heterogeneous clinical course in terms of symptom control and risk of exacerbations over time, and considered a public health issue with significant health, social and economic burden [1–3].

The World Health Survey (WHS) in 2012 revealed that global prevalence rates of doctor diagnosed asthma and clinical/treated asthma in adults were 4.3% (ranging from 0.2% in China to 21.0% in Australia, *2.06% in Türkiye*) and 4.5% (ranging from 1.0% in Vietnam to 21.5% in Australia, *2.11% in Türkiye*), respectively [4]. The observational SNAPSHOT study conducted across five countries in the Gulf cluster in 2018 revealed the adjusted prevalence of asthma to be 4.4% in the general adult population in Türkiye [5].

Anti-inflammatory maintenance treatment with inhaled corticosteroids (ICS) is the mainstay treatment in asthma, while as-needed use of short-acting β2-agonists (SABAs) for symptom relief, with or without daily maintenance treatment, has been reinforced by long-standing treatment guidelines until recently [3, 6, 7]. SABAs are rapid-onset bronchodilators effective only on symptom relief, lacking the anti-inflammatory properties of ICS [8–11]. However, due to natural tendency of patients to rely on SABAs upon symptom worsening, often without concomitant ICS, the widespread SABA overreliance and ICS underuse have become increasingly recognized problems that place patients at risk of untreated underlying inflammation and exacerbations [12–15]. Hence, using SABA-only approach without a concomitant ICS is considered a risk factor for exacerbations, while SABA overuse and the relative underuse of ICS is considered to be one of the underlying reasons for preventable asthma exacerbations and deaths [15–19].

Accordingly, as a population-level risk reduction strategy, the Global Initiative for Asthma (GINA), following the 2019 updates, no longer supports the SABA-only therapy in mild asthma, and instead now recommends low-dose ICS-formoterol in all adults and adolescents with asthma, delivered by the preferred reliever at GINA steps 3–5 for patients who are prescribed ICS/formoterol maintenance therapy, or by the symptom-driven (as-needed) reliever in mild asthma (GINA steps 1–2) [3, 6, 7, 20].

The studies in the context of SABA use IN Asthma (SABINA) program, including SABINA I (in the UK), SABINA II (in Europe, Canada and Israel), and SABINA III (globally across 24 countries) studies, indicated that SABA overuse is common (~ 30%) among asthma patients across several countries [15, 21–23]. SABA overuse was associated with poor clinical outcomes across a broad range of countries, healthcare settings and asthma severities in the global SABINA III study [23]. The data from the Türkiye arm of the SABINA III study also indicated SABA overuse in nearly a quarter of the patients [24], providing support for initiatives to improve asthma morbidity by reducing SABA overreliance [15, 23].

Better clinical control of asthma is a feasible target to decrease economic burden of asthma, particularly in terms of cost savings related to direct costs, as reported in previous cost of illness studies of asthma including those from Türkiye [24–28]. Nonetheless, while several studies indicated the relationship between SABA overuse and adverse clinical outcomes (i.e., poor asthma control, exacerbations and mortality) along with the consequent increase in healthcare resource use [15, 17, 18, 21–24], there is limited number of studies specifically addressing the cost burden of SABA overuse at a national level [29, 30].

Therefore, this cost of illness study aimed to determine the economic burden of SABA overuse in asthma patients in Türkiye from payer perspective with respect to the updated GINA 2022 treatment recommendations [3, 6, 7].

## Methods

### Design

This cost of illness study was based on identification of cost burden (severe exacerbations and drug cost) related to SABA overuse in Türkiye. Patients were categorized by their SABA prescriptions with over-prescription defined as prescribing ≥ 3 canisters of SABA per year. The economic burden (costs related to the drug use and severe exacerbations) was compared in subgroups of overall (≥ 0 canisters/year) vs. GINA-recommended (0–2 canisters/year, hypothetical population) SABA use, as well as in subgroups of appropriate use (0–2 canisters/year, real population) vs. overuse (≥ 3 canisters/year) of SABA with extrapolation of SABINA Türkiye data to the overall Türkiye asthma population. SABINA Türkiye (Türkiye arm of the SABINA III study) was an observational, cross-sectional study including 579 patients (aged ≥ 12 years) with asthma from 24 centres across Türkiye between September 2019 and January 2020 [24].

The use of two different categorizations of comparison (overall use vs. GINA- recommended use, and appropriate use vs. overuse) for the cost analysis was based on the different numbers of mild asthma and moderate-severe asthma patients assigned to the “0–2 canisters/year” SABA use in the GINA-recommended (*n* = 525,034 and *n* = 2,509,845, respectively) and the appropriate use (*n* = 483,557 and 1,809,598, respectively) subgroups. In the first category (overall use vs. GINA-recommended use), the number of patients with mild asthma (*n* = 525,034) and moderate-to-severe asthma (*n* = 2,509,845) was used with further assumption of all patients to be in the “overall use” group or in the “GINA-recommended use” group. In the second category (appropriate use vs. overuse), since there is no overall-use group, the number of patients with mild asthma (*n* = 525,034) and moderate-to-severe asthma (*n* = 2,509,845) were subjected to a further calculation and distributed using the relevant proportions of appropriate use (0–2 canisters/year; *n* = 483,577 for mild asthma; *n* = 1,809,598 for moderate-severe asthma) and overuse (≥ 3 canisters/year; *n* = 41,477 for mild asthma; *n* = 700,247 for moderate-to-severe asthma). Hence, while the total number of patients evaluated in the two comparison categories was the same, the number of mild-asthma patients and moderate-severe asthma patients in the “0–2 canisters/year” subgroup differs between the two categorizations due to use of different calculations with varied assumptions.

### Cost analysis

Direct medical cost was calculated based on cost items related to severe exacerbations (healthcare resource use during emergency or outpatient admission and hospitalization) and drug costs from payer perspective, using the prices of the public payer “Social Security Institution (SSI)” in Türkiye in accordance with the Health Implementation Notification by SSI (March 2023) [31]. For drugs, the SABINA Türkiye data [24] on SABA monotherapy and add-on treatment rates were used for extrapolation, while the costs related to healthcare resource use during emergency or outpatient admission and hospitalization for asthma exacerbation were calculated based on previously reported utility rates in cost-of-illness and SABINA Türkiye studies [24, 25, 27, 28] (Table 1). Monetary results were converted by using CBRT March 2023 effective buying rate (20.27 TL/€). Direct non-medical costs of different origin (i.e., transfers of patient and caregivers for examinations and/or hospitalization, home care) and indirect costs were not included in the cost analysis.

**Table 1:** Unit costs related to healthcare resource utilization and SABA use by the course of severe exacerbation and the disease severity

		Healthcare resource utilization					
	%	Hospitalization				Emergency care	Outpatient care
		General ward		Intensive care unit			
		LOS	Unit cost (€)	LOS	Unit cost (€)	Unit cost (€)	Unit cost (€)
Severe exacerbation							
Controlled	43.7	2	18.11	0	45.72^a^ 62.95^b^	56.42	3.67
Partially controlled	32.5	2		2			
Uncontrolled	23.8	3		4			
Average severe exacerbation cost (€)							160.23
	SABA use, %						
Asthma severity	Monotherapy				Add-on		
Mild asthma	18.4				40.8		
Moderate-severe asthma	3				65.5		
Total	5.7				61.5		
Unit drug cost /canister (€)							3.33

*LOS* Length of stay

^a^first and last days

^b^fixed

### Statistical analysis

Descriptive statistics were used to summarize results on practice patterns for the asthma management in adult patients. Expenses related to management of adult asthma were the main cost-analysis related parameter of the study. Cost model was based on the following equation: “Cost = ∑ (Frequency; %) X (Unit price; TL) X (patient ratio; %)”.

## Results

### Reference data for asthma prevalence and exacerbations in Türkiye

Given the 2022 Turkey population of individuals over 18 years of age (*n* = 68,974,525) and asthma prevalence of 4.4% [5], overall number of adult asthma patients in Türkiye was estimated to be 3,034,879. Accordingly, a total of 3,034,879 adult asthma patients including 525,034 (17.3%) mild asthma patients (0.3 severe exacerbations per year, GINA Step 1: 208,963 [39.8%] patients, GINA step 2: 316,071 [60.2%] patients) and 2,509,845 (82.7%) moderate-severe asthma patients (1.3 severe exacerbations per year, GINA Step 3: 559,696 [22.3%] patients, GINA step 4: 981,350 [39.1%] patients, GINA step 5: 968,799 [38.6%]) comprised the study population, via estimations extrapolated from the Türkiye arm of the global SABINA III study [23, 24] (Table 2).

**Table 2:** Reference data for the asthma prevalence and SABA prescription in relation to severe exacerbation frequency in mild and moderate-to-severe asthma patients in Türkiye

	**Adult asthma patients (*** n* = **3,034,879)**					
	**Mild asthma**			**Moderate-severe asthma**		
	**%**		**n**	**%**		**n**
**Asthma prevalence**	17.3		525,034	82.7		2,509,845
**Patients with severe exacerbations**	17.3		90,831	52.6		1,320,179
**Annual per patient SEF**	0.3			1.3		
**GINA Step**						
Step 1	39.8		208,963	-		-
Step 2	60.2		316,071	-		-
Step 3	-		-	22.3		559,696
Step 4	-		-	39.1		981,350
Step 5	-		-	38.6		968,799
	**Mild asthma**			**Moderate-severe asthma**		
	**Patients**		**Annual SEF**	**Patients**		**Annual SEF**
	%	n		%	n	
**SABA prescription (canister/year)**						
0–2 canisters	92.1	483,557	27,634	72.0	1,809,598	428,189
3–5 canisters	3.4	17,851	38,687	13.8	346,358	599,465
6–9 canisters	1.1	5,775	42,003	9.0	225,886	650,847
10–12 canisters	3.4	17,851	49,188	3.9	100,394	762,176
≥ 13 canisters	0.0	0	0	1.1	27,609	822,123
**Total**		525,034	157,512		2,509,845	3,262,800

*SEF* Severe exacerbation frequency

### Reference data for *SABA* prescription in relation to exacerbation frequency in Türkiye

Patients were categorized by their SABA (0, 1–2, 3–5, 6–9, 10–12 and ≥ 13 canisters per year) prescriptions in the 12 months prior to the study visit with over-prescription defined as prescribing ≥ 3 canisters of SABA per year. Based on the increase in severe asthma exacerbation incidence rate ratio (aIRR) with increase in yearly SABA prescription reported in global SABINA III study [23] as well as data from the Türkiye arm of the global SABINA III study [24], annual severe asthma exacerbation frequency in mild and moderate-severe asthma patients were distributed according to SABA prescription subgroups. Accordingly, 483,557 mild asthma and 1,809,598 moderate-severe asthma patients with appropriate SABA use (0–2 canisters per year) were considered to have 27,634 and 428,189 severe asthma exacerbations per year, respectively, while 700,247 moderate-severe asthma patients with SABA over-prescription (≥ 3 SABA canister prescriptions per year) had 2,834,611 severe asthma exacerbations per year (Table 2).

### The cost burden of not applying GINA-recommended *SABA* use

Overall, recommended SABA use was predicted to prevent 127,505 (80.9%) of 157,512 severe exacerbations per year in mild asthma patients and 2,668,916 (81.8%) of 3,262,800 severe exacerbations per year in moderate-severe asthma patients (Table 3).

**Table 3:** The direct (drug and severe exacerbations) costs in subgroups of overall vs. GINA-recommended (0–2 canisters per year) SABA use: Burden of not applying recommended use

	**SABA usage**		**Burden of not applying recommended use**		
	**Overall (≥ 0 canisters/year)**^**a**^	**GINA-recommended (0–2 canisters/year)**^b*^	**SABA canisters**	**Severe exacerbation frequency**	**Cost burden (€)**
**Mild asthma**					
Number of patients (n)	525,034	525,034		-	
Severe exacerbation frequency	157,512	30,007		127,505	
**Cost of severe exacerbation (€)**	**25,238,605**	**4,808,109**			**20,430,496**
Number of SABA canisters	669,420	420,553	248,867		
**SABA drug cost (€)**	**2,230,850**	**1,401,498**			**829,352**
**Moderate-severe asthma**					
Number of patients (n)	2,509,845	2,509,845		-	
Severe exacerbation frequency	3,262,800	593,884		2,668,916	
**Cost of severe exacerbation (€)**	**522,807,882**	**95,159,749**			**427,648,133**
Number of SABA canisters	3,502,028	1,340,710	2,161,318		
**SABA drug cost (€)**	**11,670,547**	**4,467,931**			**7,202,616**
**Total cost burden (€)**					**456,110,597**

SABINA III Türkiye data on ^a^overall SABA use (≥ 0 canisters/year) and ^b^GINA-recommended SABA use (0–2 canisters/year, hypothetical population) were extrapolated to overall Türkiye population ^*^GINA-recommended term refers to a hypothetical population of 0–2 SABA canister users (not a population who actually use 0–2 canisters per year), assuming that all patients use SABA correctly and there is no overuse, which was obtained by projecting the number of patients who use 0–2 canisters per year to the total number of patients as if the entire patient population consisted of those who use 0–2 canisters per year

The annual cost burden of not applying recommended SABA use in mild asthma and moderate-severe asthma patients was calculated to be €20.43 million and €427.65 million in terms of severe exacerbations, and to be €829,352 and €7.20 million in terms of drug costs, respectively. The total annual economic burden arising from not applying recommended SABA use was estimated to be €456.11 million (Table 3).

### The cost burden of *SABA* overuse (≥ 3 canisters per year)

Overall, appropriate use vs. overuse of SABA was associated with decreased frequency of severe exacerbations per year in mild asthma (from 129,878 to 27,634, by 78.7%) and moderate-severe asthma (from 2,834,611 to 428,189, by 84.9%) patients (Table 4).

**Table 4:** The direct (drug and severe exacerbations) costs in subgroups of appropriate use (0–2 canisters per year) vs. overuse (≥ 3 canisters per year) of SABA: Burden of SABA overuse

	**SABA usage**		**Burden of SABA overuse**		
	**Appropriate use** **(0–2 canisters per year)**^**a**^	**Overuse** **(≥ 3 canisters per year)**^**b**^	**SABA canisters**	**Severe exacerbation frequency**	**Cost burden (€)**
**Mild asthma**					
Number of patients (n)	483,557	41,477	-		
Severe exacerbation frequency	27,634	129,878		102,244	
**Cost of severe exacerbation (€)**	**4,427,783**	**20,810,581**			**16,382,798**
Number of SABA canisters	358,336	311,083	-47,253^*^		
**SABA drug cost (€)**	**1,194,159**	**1,036,688**			-157,471^*^
**Moderate-severe asthma**					
Number of patients (n)	1,809,598	700,247	-		
Severe exacerbation frequency	428,189	2,834,611		2,406,422	
**Cost of severe exacerbation (€)**	**68,609,958**	**454,197,924**			**385,587,965**
Number of SABA canisters	1,133,195	4,570,426	3,437,231		
**SABA drug cost (€)**	**3,776,385**	**15,230,995**			**11,454,610**
**SABA drug cost-overall (€)**					**11,297,139**
**Total cost burden (€)**					**413,267,903**

SABINA III Türkiye data on ^a^appropriate use (0–2 canisters per year; real population) and ^b^overuse (≥ 3 canisters per year) of SABA were extrapolated to overall Türkiye population

^*^a negative number of SABA canisters and negative cost burden for mild asthma are related to considerably (11.7 times) lower number of patients using ≥ 3 canisters of SABA (41,477) than the number of patients using 0–2 canisters of SABA (483,557) in the mild asthma sample

Considering appropriate use vs. SABA overuse subgroups, SABA overuse in mild and moderate-severe asthma patients was estimated to yield an additional annual cost of €16.38 million and €385.59 million, respectively in terms of severe exacerbations, and a total €11.30 million additional drug cost. The overall annual economic burden arising from SABA overuse was estimated to be €413.27 million (Table 4).

## Discussion

In this cost of illness study, not applying the GINA-recommended SABA use was found to be associated with an estimated cost burden of €456.11 million per year overall, including the additional costs related to severe exacerbations (€20.43 million in mild asthma and €427.65 million in moderate-severe asthma) and the drug cost (€829,352 in mild asthma and €7.20 million in moderate-severe asthma). The total cost burden arising from SABA overuse was estimated to be €413.27 million per year overall, comprising the additional costs related to severe exacerbations (€16.38 million in mild asthma and €385.59 million in moderate-severe asthma) and a total €11.30 million of drug cost. Accordingly, the cost burden attributable to SABA overuse was particularly relevant for the moderate-to-severe asthma (95.3% of total cost burden, 93.8% were related to severe exacerbations) and the healthcare resource utilization related to severe exacerbations was the key cost driver.

Indeed, asthma has been recognized as a priority disorder in government health strategies due to high prevalence and significant economic burden to health systems with the total annual cost estimated to be $18 billion in the USA, €4.3 billion in European countries, and €2.3 billion in Türkiye [3, 26, 27, 32]. Representing one of the few studies on the cost burden of SABA overuse in asthma patients, our results support the increase in healthcare costs in patients with high- versus low-SABA prescriptions, emphasizing the likelihood of achieving potential cost savings in asthma care by adopting the latest evidence-based GINA recommendations regarding the reductions in SABA usage [29, 30].

The SABINA study from Spain involving 39,555 patients revealed the SABA overuse (≥ 3 canisters/year) in 28.7% of patients, and the association of SABA overuse vs. the recommended use (< 2 canisters/year) with a significant increase in health care costs per patient and year (€5,702 vs €1,917) [29]. In addition, the hospital admissions (40.6%), associated medication (23.9%), and productivity loss (13%) were the major cost drivers, while underuse of ICS (vs. recommended use) was also associated with a higher exacerbation rate (1.2 vs 0.6) and an increase in health care costs (€4,116 vs €2,902) [29].

In the SABINA I study from UK, SABA overuse (≥ 3 canisters/year) was noted in 94,544 (51%) patients, and found to increase with increasing asthma treatment step (from 28% in the group with no ICS to 77% in the group with high-dose ICS ± additional therapies) [30]. The total annual average costs (healthcare resource utilization and medication) were found to be 52% higher in the high- versus low-SABA group (£2,256,091/1,000 versus £1,480,640/1,000 patients/year), while the extrapolation of findings to an estimated 4.5 million patients with asthma in the broader UK population revealed that higher SABA prescriptions translated to an estimated additional cost of £108.5 million per year overall [30]. In addition, while the patients in the high-SABA group (£474,794/1,000 patients/year) had 69% greater annual average healthcare resource utilization costs than the low-SABA group (£280,280/1,000 patients/year), the medication costs accounted for most of the asthma-related costs, and overall differences in healthcare resource utilization were primarily driven by non–exacerbation-related healthcare resource utilization [30].

In a previous cost of illness study, per patient annual direct medical cost of adult asthma in Türkiye was estimated to be $1,371.20 with hospitalizations ($811.39, 59.2%) as the main cost driver, followed by drug treatment/equipment ($252.42, 18.4%) and co-morbidities/complications ($151.46, 11.1%) [25]. In another cost of illness study, the total per patient annual direct medical cost of severe asthma in Türkiye was calculated to be $4,369.76 with drug treatment/equipment ($ 2,289.63, 52.4%) as the main cost driver, followed by hospitalizations/interventions ($ 1,154.55, 26.4%) and co-morbidities ($ 665.39, 15.2%) [27]. In another cost of illness study on the direct cost of per asthma exacerbation in Türkiye, asthma exacerbations that were associated with hospitalization accounted for majority of the total costs of exacerbations, indicating the major contribution of inpatient follow-up to healthcare resource utilization cost [28].

Nonetheless, the detection of hospitalization in almost 80% of asthma patients is considered highly suggestive of unnecessary and inappropriate hospitalization since asthma attack was mild to moderate in more than 75% of these patients [28]. Besides, the 2018 Global Asthma Report revealed that Türkiye reported the highest rate of hospital admissions for asthma (all ages) among 30 European countries between 2011 and 2015 [33].

Thus, the inappropriate hospitalization for asthma exacerbations may account for the higher estimated cost burden of SABA overuse in our study than reported in the UK asthma population (€413.27 million vs. £108.5 million per year, respectively) despite the lower rate of SABA overuse in our study (24.4% vs. 51%, respectively) [30], emphasizing the likelihood of a cost-saving with appropriate hospitalization and better asthma control via effective implementation of best practice [25, 27, 28, 34–37].

Considering the prevalence of SABA overuse, the SABINA I and II studies have shown that SABA over-prescription (≥ 3 canisters per year) is common (33% overall, ranged from 9% in Italy to 38% in UK) across European countries [15, 22]. The global SABINA III study across 24 countries also revealed that 38% of patients were prescribed ≥ 3 SABA canisters and 18.0% purchased over-the-counter (OTC) SABA [23]. The data from SABINA III studies in individual countries such as Russia (37%), Asian countries (15.9% in Taiwan to 47.4% in Malaysia), Middle Eastern countries (47.1%) and Gulf region (58.5%) also revealed the high prevalence of SABA overuse [18, 23, 38–40].

In the analysis of Turkish patients included in the SABINA III study, SABA overuse was noted in 23.9% of patients despite all patients being treated by specialists and most receiving fully reimbursed healthcare, emphasizing the need for improved clinical practice aligned with the latest evidence-based recommendations [24]. Additionally, OTC SABA usage by 10.2% of patients (≥ 3 canisters by 27.1%) is considered to indicate SABA over-reliance with willingness of patients to self-manage worsening asthma symptoms [24, 41–43].

In the present analysis, the estimated prevalence of SABA overuse was ~ 24% overall, including 27.9% of moderate-severe asthma patients and 8.0% of mild asthma patients, indicating ~ 3.5 times greater prevalence of SABA overuse in those with moderate-severe versus mild asthma. Likewise, in the UK (SABINA program), SABA overuse was found to be greater in individuals with moderate-to-severe asthma versus individuals with mild asthma (58% versus 27%, respectively), whereas SABA overuse was similar in mild (9–32%) and moderate-to-severe (8–31%) asthma in the other European countries [15, 22]. Also, a Polish study of pharmacy prescription records of 91,673 adult patients indicated that 29–37% of patients with asthma were prescribed at least three SABA canisters per year, while the greater SABA overuse in moderate-to-severe asthma patients (up to 58%) than in mild asthma patients is suggested to indicate likelihood of a poorer asthma control in patients with moderate-severe asthma despite receiving maintenance ICS [44].

Nonetheless, our findings revealed that appropriate SABA usage was effective in decreasing the annual severe exacerbation frequency, similarly in mild and moderate-severe asthma patients (by 78.7% and 84.9%, respectively). Likewise, SABINA study in Spain also showed that SABA overuse was associated an increase of 1.52 times in the estimated number of exacerbations/years compared with patients who were prescribed ≤ 2 SABA canisters [45].

In fact, increased frequency of annual exacerbations via SABA overuse vs. recommended dosing is considered important not only for the related cost increment but also for the related poor clinical outcomes, such as increased risk of poor asthma control, severe exacerbations and hospitalization or emergency department visits as well as the risk of asthma-related death, providing support for initiatives to improve asthma morbidity by reducing the progressive SABA overreliance [11, 17, 22, 23, 29, 41, 46–50]. The prevention of SABA overreliance with improved adherence to the latest evidence-based recommendations and patient education is considered to be of critical importance to achieve cost-saving by reducing the number of exacerbations and hospitalizations and a reduced asthma morbidity [3, 6, 7, 11, 25, 27, 29, 51, 52].

Certain limitations to this study should be considered. First, lack of data on indirect costs or intangible costs of illness seems to be the major limitation of the present study which likely to result in a downward bias in our estimates of the economic burden of SABA overuse. Second, given that SABA prescriptions were used as a proxy for potential use in different everyday scenarios, SABA prescriptions may not reflect actual SABA use/overuse. Third, given the inclusion of only severe exacerbation costs and SABA costs in the present analysis, much higher costs can be expected considering the overall exacerbations and entire medications. Fourth, while the prevalence of SABA overuse in the Türkiye arm of the SABINA III study was reported to be 23.9%, this is optimistic data limited to tertiary care practice. Finally, while a cost-of-illness study gives a perspective in a population, it does not reflect what is happening with the individual patient or family unit.

## Conclusion

In conclusion, the estimated total economic burden arising from not applying recommended SABA use (€456.11 million) and the SABA overuse (€413.27 million) with respect to the updated GINA 2022 treatment recommendations in the present cost analysis indicates the substantial cost burden of SABA overuse to the Turkish National Health System, corresponding up to 26% of the total direct cost of asthma reported in our country. Our findings indicate approximately 3.5 times greater prevalence of SABA overuse in patients with moderate-severe asthma than in those with mild asthma, while the cost burden attributable to SABA overuse was particularly relevant for the moderate-to-severe asthma and the healthcare resource utilization related to severe exacerbations. Nonetheless, appropriate SABA usage was effective in decreasing the annual severe exacerbation frequency by > 80% in our asthma patients, regardless of disease severity. Initiatives with collaborative efforts between policymakers and healthcare professionals that address reducing the progressive SABA overreliance with improved adherence to the latest evidence-based recommendations are necessary to reduce the asthma morbidity and the cost burden related to this public health concern.

## Data Availability

No datasets were generated or analysed during the current study.

## Abbreviations

aIRR: Asthma exacerbation incidence rate ratio
CBRT: Central Bank of the Republic of Türkiye
GINA: Global Initiative for Asthma
ICS: Inhaled corticosteroids
LOS: Length of stay
OTC: Over-the-counter
SABINA: SABA use IN Asthma
SABAs: Short-acting β2-agonists
SEF: Severe exacerbation frequency
SSI: Social Security Institution
UK: United Kingdom
WHS: World Health Survey
